# Metabolic and cardiovascular disease risk for Zimbabwean men with prostate cancer receiving long-term androgen deprivation therapy

**DOI:** 10.21203/rs.3.rs-3723949/v1

**Published:** 2023-12-12

**Authors:** Tinashe Mazhindu, Ntokozo Ndlovu, Margaret Z. Borok, Shingirirai Meki, Albert Nyamhunga, Edward P. Havranek, Elizabeth R. Kessler, Thomas B. Campbell, Thomas W. Flaig

**Affiliations:** University of Zimbabwe; University of Zimbabwe; University of Zimbabwe; University of Zimbabwe; University of Zimbabwe; University of Colorado Anschutz; University of Colorado Anschutz; University of Colorado Anschutz; University of Colorado Anschutz

**Keywords:** Androgen deprivation therapy, African men, prostate cancer, metabolic syndrome

## Abstract

**Introduction:**

Prostate cancer is a leading cause of cancer-related mortality in the majority of sub-Saharan Africa region countries. Androgen deprivation therapy (ADT) is effective treatment, however ADT is associated with complications including metabolic syndrome and cardiovascular disease. Although cardiovascular disease is a leading cause of mortality among prostate cancer patients, there is limited information on ADT impact on metabolic syndrome and cardiovascular disease risk among Africans. An observational prospective cohort study was carried out in Harare, Zimbabwe. Prostate cancer patients due to be initiated on ADT (medical or surgical) were assessed for metabolic syndrome and a 10-year Atherosclerotic Cardiovascular Disease (ASCVD) 10-year risk probability score was done before ADT and followed up to 9 months.

**Results:**

17 black Zimbabwean men were enrolled with a median age 72 years. Most participants (59%) had stage IV disease and 75% opted for surgical castration. At enrolment 23.5% had metabolic syndrome and this increased to 33% after 9 months of ADT. Baseline ASCVD risk was in the high risk category for 68.8% of participants and remained above 50% after 9 months of ADT. In this cohort, there is a 10% absolute increase in metabolic syndrome prevalence amongst African men with prostate cancer within 9 months of ADT initiation.

## Introduction

Prostate cancer is a leading cause of cancer-related mortality in men globally, including most sub-Saharan African (SSA) countries like Zimbabwe.(1, 2) Due to weak health systems and lack of prostate cancer screening in SSA countries, there is late-stage diagnosis, treatment delays, and overall poor outcomes for patients.(3) Prostate cancer mortality is estimated to increase by 117% from 2020 to 2040 in Africa. (4) The Zimbabwe National Cancer Registry annual reports shows that the incidence of prostate cancer has risen over the last 20 years, and overtaken Kaposi sarcoma incidence to become second only to cervical cancer.(2, 5) Prostate cancer treatment often includes multimodal interventions including and over of all patients receive androgen-deprivation therapy (ADT) at some point globally.(6–9)

The median age for prostate cancer diagnosis globally is 66 years and elderly patients often have comorbidities that need to be considered in prostate cancer case management. (10) ADT independently is associated with several metabolic complications, including abdominal obesity, poor blood sugar control, dyslipidemia, metabolic syndrome, and cardiovascular disease (CVD). (11) CVD is a leading cause of morbidity and mortality among prostate cancer patients due to various factors including age-demographic and treatment factors. Evidence showing that there is an increase in CVD risk in men with prostate cancer on ADT has been observed in randomized studies and population based studies with some researchers reporting a 40% increase in non-fatal CVD.(12) Men with prostate cancer have a higher incidence of CVD compared to men with no history of prostate cancer.(13) CVD risk can be assessed by various methods including the American College of Cardiology (ACC)/American Heart Association (AHA) Atherosclerotic cardiovascular disease (ASCVD) 10-year risk calculation and the incidence of metabolic syndrome. Metabolic syndrome is a cluster of biochemical and physiological abnormalities which collectively raise the risk of ischemic heart disease, diabetes mellitus, stroke, and other health complications.(14) Male hypogonadism is an independent risk factor for metabolic syndrome.(11)

Despite the growing burden of prostate cancer in Africa, there are limited studies on metabolic syndrome and CVD risk trends amongst African prostate cancer patients. ADT has been associated with poorer overall survival among African American men with favorable-risk prostate cancer.(15) Studies in Africa have reported a cross-sectional increase in CVD risk factors in ADT recipients in Nigeria and a longitudinal study following South African men observed a similar pattern.(16, 17) This prospective cohort study was conducted to evaluate CVD risk using both metabolic syndrome prevalence and ASCVD 10-year risk calculation in African men on ADT.

## Methodology

### Study Design & setting

A prospective cohort study was conducted at the Parirenyatwa Group of Hospitals, Harare, Zimbabwe. Incident prostate adenocarcinoma patients were enrolled prior to initiating ADT between 1 March 2021 and 30 September 2021 after obtaining informed consent.

### Data collection

Participants were assessed for disease status, metabolic syndrome and CVD risk at baseline, and thereafter at 3, 6 and 9 months after initiating ADT. This was done by measurement of waist and hip circumference, blood pressure, fasting glucose, triglycerides, high-density lipoprotein (HDL) and total cholesterol. Other tests done included prostate specific antigen (PSA), renal function, full blood count, and HIV testing. A blood sample was also biobanked for future utility at the African Institute of Biomedical Science & Technology (AiBST) biobank Zimbabwe. Treatment for prostate cancer, including ADT, and co-morbidities was provided by local standard of care and directed according to the treating physicians’ choice.

### Cardiovascular disease risk estimation

*Metabolic syndrome* was determined by the NCEP ATP III definition outlined in Table 1 below:

*Cardiovascular risk assessment*: Cardiovascular risk was calculated using the ACC publicly accessible online **ASCVD Risk Estimator** https://tools.acc.org/ascvd-risk-estimator-plus/#!/calculate/estimate/ and risk scores categorized into low (< 5%), borderline (5-<7.5%), intermediate (7.5-<20%), and high (≥ 20%).(18) The study was approved by the Medical Research Council of Zimbabwe (MRCZ/A/2623).

Variables, such as age and plasma metabolite measurements, were calculated and analyzed as means, medians and interquartile range. Qualitative variables, such as cancer stage, performance status and co-medications were collected and presented as absolute numbers and percentages. The risk factors for cardiovascular disease were recorded and analyzed as proportions of the total enrolled participants or total reviewed participants for each 3 monthly interval.

## Results

### Study population

Seventeen black Zimbabwean men with prostate cancer were enrolled into the study with a median age of 72 years (IQR 65–75). Most of the participants lived in a rural area (64.7%) and none had a history of tobacco smoking. All participants had locally advanced or metastatic disease with a distribution of 7 (41%) with clinical stage III and 10 (59%) with stage IV disease. The median plasma PSA level at enrollment was 162ng/ml (IQR 52–379). Sixteen participants commenced androgen deprivation therapy (ADT) as planned and the interval between the decision to start ADT and actual undertaking ranged between 0–36 days (mean 7.8 days). One patient later declined to start ADT treatment. Surgical bilateral orchidectomy was performed in 75% and 25% started Zoladex ^®^ S.C 10.8mg 3 monthly. (Table 2).

### Cardiovascular disease and risk profile at enrollment

Twelve participants (70.6%) were on treatment for hypertension at the time of prostate cancer diagnosis. Two participants had a previous diagnosis of diabetes mellitus and were both on treatment with oral hypoglycemics. Out of the 15 participants with no known history of diabetes mellitus, 6 (40%) had elevated fasting glucose levels above 5.6mmol/l at baseline. Four participants (23.5%) had metabolic syndrome. Baseline 10-year atherosclerotic cardiovascular disease (ASCVD) risk score showed that 68.8%, 31.2%, 0% and 0% had a high risk, intermediate risk, borderline risk and normal risk classification respectively. (Table 3)

### Study cohort follow up

**Figure 1:**
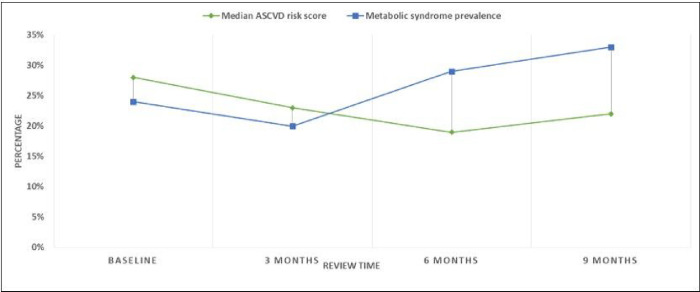
Metabolic syndrome prevalence and atherosclerotic cardiovascular disease risk score among study participants at baseline enrollment, 3months, 6 months and 9 months after ADT commencement.

All participants except one had a sustained biochemical disease response observed up to 9 months on ADT. A single participant had biochemical failure observed 9 months after surgical castration. The median ASCVD score trend showed an initial reduction up to 6 months follow up and increased between the 6^th^ month and 9^th^ month review. All participants had an ASCVD risk score >7.5% throughout the study. The prevalence of metabolic syndrome showed an initial reduction from 23.5% at baseline assessment to 20% at 3 months, thereafter, it increased to 33.3% at 9 months shown in Figure 1.

## Discussion

All patients presented with advanced disease: 59% had stage IV disease and the remainder had stage III. The median age for the cohort was also older than the global median for prostate cancer. This could be due to lack of prostate cancer screening and in addition age as an independent risk factor for CVD.

The prevalence of metabolic syndrome was comparable to other studies which ranged from 22–42% with the higher percentage being observed in elderly patients.(19, 20) Metabolic syndrome risk increases with age and ethno-racial differences have been observed. The follow up period after ADT commencement showed a slight initial reduction in metabolic syndrome prevalence followed by a consistent increase. We postulate that one cause for the slight initial reduction was due to identification of abnormalities such as high fasting blood sugar, high cholesterol and hypertension which are CVD risk factors at enrollment and the study allowed for the treating physicians to actively treat these conditions. The observed increase at the 6th and 9th month reviews ultimately resulted in 1 out every 3 participants having metabolic syndrome. This observed increase in metabolic syndrome is consistent with the previously known phenomenon of increased metabolic syndrome development due to ADT induced increase in insulin resistance, increased fasting blood glucose levels and fasting triglycerides levels. (11, 18) This phenomenon has been observed in African men on ADT showing cholesterol, triglyceride, fasting blood glucose level and abdominal circumference increase with the background that African men generally have a higher risk of CVD.(21) Population-based analysis suggest that metabolic syndrome could account for about 20% of all strokes.(20)

Using the ASCVD 10-year risk calculator, all 17 study participants had intermediate or higher risk score findings. This scoring system is designed to calculate the risk of CVD like myocardial infarction and cerebrovascular accidents. At enrolment 68.8% had a high risk score and the baseline median ASCVD 10-year risk score was 29.5% which is in the intermediate risk category. During follow up, an initial reduction in median score at the the 3rd month and 6th month review was observed and an upward trend at the 9th month review. This observed initial reduction could be for the same reason as observed in metabolic syndrome. Metabolic syndrome on its own is a risk factor for abnormal ASCVD 10-year risk findings. At no time in the study was the median ASCVD risk below 20%.

Men of African ancestry have been reported to have higher rates of CVD risk in multiple studies, however the observed level of risk in this study is higher than rates reported in other studies. In this study the median age of participants was 72 years, which is 6 years older than the global median age for new prostate cancer diagnosis. (22) The pattern of ASCVD risk and metabolic syndrome prevalence observed over 9 months is consistent with the trend of CVD risk associated with ADT in elderly men.(12, 23) The recommended management of patients with significant risk for CVD includes lifestyle modification and pharmacological intervention. Recognition of this risk and optimizing patient care can yield better morbidity and mortality outcomes.

Limitations to this study. This study was conducted during the COVID-19 pandemic national lockdown period in Zimbabwe. It was resultantly limited in sample size, study follow up period of just 9 months and, use of ASCVD 10-year risk calculator which is not validated in African populations. As a result definitive inference or more detailed statistical analysis were not possible. Despite the limited sample size, the feasibility of incorporating CVD risk monitoring into ADT clinic care is shown.

## Figures and Tables

**Table 1:** Metabolic syndrome criteria determined by the NCEP ATP III definition

NCEP: ATP III Diagnostic Criteria for Metabolic Syndrome (Presence of 3 or more of these components)	
Abdominal obesity: Increased waist circumference	Men: ≥40 inches (102 cm)
Elevated triglycerides	≥150 mg/dL (1.7 mmol/1) or drug treatment for elevated triglycerides
Reduced HDL-Cholesterol (HDL-C)	Men: <40 mg/dL(<1.03mmol/L); women: <50 mg/dL (<1.29mmol/L)
Elevated blood pressure	≥130/85 mm Hg or drug treatment for elevated blood pressure
Elevated fasting glucose	≥100 mg/dL (>5.6mmol/L) or drug treatment for elevated glucose

**Table 2:** Demographic and clinical characteristics of study participants at enrolment.

Characteristic	N (%)
Smoking status	
Smoker	0
Non-smoker	17 (100)
Current alcohol use status	
Alcohol use	5 (29.4)
No alcohol use	12 (70.6)
Employment status	
Employed	1 (5.9)
Unemployed	16 (94.1)
Primary residence	
Urban	6 (35.3)
Rural	11 (64.7)
Highest level of education	
Primary	10 (58.8)
Secondary	5 (29.4)
Tertiary	2 (11.8)
Marital status	
Married	15 (88.2)
Widowed	2 (11.8)
HIV status	
Positive	2 (11.8)
Negative	15 (88.2)
Baseline body mass index	
< 18.5	1 (6)
18.5–24.9	12 (71)
25–29.9	4 (24)
30–34.9	0
35–39.9	0
>40	0
ECOG status	
0	7 (41.2)
1	8 (47.1)
2	2 (11.8)
WHO prostate cancer grade group	
1	1 (5.9)
2	2 (11.8)
3	7 (41.2)
4	3 (17.7)
5	4 (23.5)
AJCC group stage	
1	0
2	0
3	7 (41)
4	10 (59)
Androgen derivation therapy	
Surgical	12 (75)
Medical	4 (25)

Key: AJCC = American Joint Committee on Cancer; ECOG = Eastern Cooperative Oncology Group; HIV = Human immunodeficiency virus; WHO = World Health Organisation

**Table 3:** Cardiovascular disease risk factor distribution at enrolment into the study

Characteristic (N=17)	N (IQR or %)
**Median abdominal circumference [cm]**	83 (IQR 80 – 91)
**Median systolic blood pressure [mmHg]**	155 (IQR 143 – 161)
**Median diastolic blood pressure [mmHg]**	84 (IQR 76 – 93)
**Median triglyceride levels [mmol/l]**	0.95 (IQR 0.84 – 1.52)
**Median total cholesterol levels [mmol/l]**	5.34 (IQR 4.51 – 5.45)
**Median HDL-cholesterol levels [mmol/l]**	1.18 (IQR 0.99 – 1.35)
**Median fasting glucose levels [mmol/l]**	5.53 (IQR 5.11 – 6.99)
	
**Pre existing diabetes**	
Yes	2 (11.8%)
No	15 (88.2%)
**Pre-existing hypertension**	
Yes	12 (70.6%)
No	5 (29.4%)
**Elevated fasting glucose**	
Yes	8 (47.1%)
No	9 (52.9%)
**ASCVD risk (%)**	
Low (<5)	0
Borderline (5–<7.5)	0
Intermediate (7.5–<20)	5 (31.2%)
High (≥20)	11 (68.8%)
Median ASCVD risk	28.6%
**Metabolic syndrome**	
No	13 (76.5%)
Yes	4 (23.5%)

## Data Availability

The data from the study is available upon request through the corresponding author. Anonymized data is available for sharing.
